# Novel agents and evolving strategies for anemia management in lower-risk myelodysplastic syndromes

**DOI:** 10.1007/s44313-025-00099-x

**Published:** 2025-09-30

**Authors:** Junshik Hong

**Affiliations:** 1 Department of Internal Medicine, Seoul National University College of Medicine, Seoul National University Hospital, Seoul, Republic of Korea; 2 Cancer Research Institute, Seoul National University College of Medicine, Seoul, Republic of Korea

**Keywords:** Myelodysplastic syndrome, Anemia, Luspatercept, Imetelstat, Erythropoiesis

## Abstract

Recent developments in the treatment of lower-risk myelodysplastic syndromes have focused on improving anemia management, which remains a major clinical challenge. Erythropoiesis-stimulating agents (ESAs) and lenalidomide are the standard therapies; however, their effectiveness is limited by resistance and patient selection criteria. Luspatercept, a transforming growth factor-beta superfamily ligand trap, has shown improved transfusion independence and is now considered a frontline option for a broader group of patients. Clinical trials have indicated that luspatercept provides a sustained response in several cases. Imetelstat, a telomerase inhibitor, offers an alternative for patients who do not respond to ESAs and has been shown to reduce the clonal mutation burden, suggesting possible disease-modifying effects. However, unresolved issues remain, such as the lack of predictive biomarkers to guide therapy selection, uncertainty about the optimal sequencing or combination of available treatments, and the fact that most patients eventually progress to higher-risk disease. Additionally, the real-world use of these new agents remains limited in some regions owing to issues with local introduction and reimbursement. This review summarizes recent clinical data on luspatercept and imetelstat, highlights their current limitations, and discusses areas for future research based on recent trial outcomes and evolving clinical practices.

## Introduction

Over the past two decades, oncology has undergone a transformative revolution driven by the widespread adoption of targeted therapies [1] and the emergence of immune checkpoint inhibitors (ICIs) [2]. While these advances have been particularly pronounced in solid tumors, such as melanoma and non-small cell lung cancer, where ICIs reshaped treatment paradigms, hematologic malignancies have also seen remarkable progress. Multiple myeloma stands as a prime example, with over 20 U.S. Food and Drug Agency (FDA)-approved drugs since 2000, including proteasome inhibitors (e.g., bortezomib), immunomodulatory agents (e.g., lenalidomide), CD38-targeted monoclonal antibodies (e.g., daratumumab), and chimeric antigen receptor-T therapies targeting B cell maturation agent [3].

In contrast, therapeutic innovations in myelodysplastic syndromes (MDS) have lagged, particularly in patients at high risk. The complexity of MDS biology, characterized by clonal heterogeneity, divergent mutational landscapes, and a microenvironment that favors immune evasion, has hindered the development of targeted and immune therapies [4–6]. Although hypomethylating agents (HMAs), such as azacitidine and decitabine, remain the cornerstone treatment, their efficacy is limited, with a median overall survival of 12–18 months in high-risk MDS [7, 8]. Recent approvals have focused almost exclusively on anemia correction in lower-risk (LR) MDS, with only two notable exceptions: enasidenib (an IDH2 inhibitor) [9] and oral decitabine/cedazuridine (an oral HMA alternative) [10].

This therapeutic stagnation persists despite deeper molecular characterization of MDS. Next-generation sequencing has identified recurrent mutations in *SF3B1*, *TP53*, *TET2*, and *ASXL1* [11–13]; however, translating these insights into targeted therapies remains challenging [14] because of the lack of druggable binding sites and functional redundancy in the splicing machinery [15, 16]. Furthermore, failed clinical trials combining HMAs with agents such as ICIs [17] or anti-CD47 antibodies [18] have underscored the unique resistance mechanisms in MDS compared with those in other hematologic malignancies.

Despite ongoing challenges in MDS drug development, including the absence of predictive biomarkers, inconsistent clinical trial endpoints, and a limited understanding of clonal evolution dynamics, recent FDA approvals of luspatercept [19, 20] and imetelstat [21] represent meaningful progress in addressing anemia in patients with lower-risk MDS. Although these agents primarily improve transfusion-dependent anemia, the mechanism of imetelstat as a telomerase inhibitor raises the possibility of disease-modifying effects; however, further evidence is needed to confirm this potential [22, 23].

In this review, we summarize the clinical evidence for these two newly approved anemia-correction agents and discuss the ongoing challenges and future directions that may help advance MDS treatment beyond current standards.

## Normal and dysregulated erythropoiesis in Lower-Risk Myelodysplastic Syndromes (LR-MDS)

### Early and late erythropoiesis

Erythropoiesis, the process by which red blood cells (RBCs) are produced, is a complex and tightly regulated sequence of events that can be broadly divided into early and late stages. In the early phase, hematopoietic stem and progenitor cells differentiate into erythroid progenitors such as burst-forming unit-erythroid and colony-forming unit-erythroid. This expansion is primarily driven by erythropoietin (EPO), which binds to its receptor (EPOR) and activates downstream signaling pathways, notably the JAK2/STAT5 axis, to promote cell survival and proliferation [24]. Transcription factors, including GATA1/2 and KLF1, further coordinate the expression of genes necessary for hemoglobin (Hb) synthesis and cell cycle progression, ensuring the effective production of erythroid precursors [25].

As erythropoiesis progresses to the late stage, the maturation of proerythroblasts into reticulocytes becomes increasingly influenced by the modulatory transforming growth factor-beta (TGF-β) superfamily of cytokines, such as GDF11 and activins. These ligands interact with specific type II receptors (ACVR2A/B), leading to the activation of SMAD2/3 signaling. This pathway exerts an inhibitory effect on erythroid maturation, inducing apoptosis and causing maturation arrest, which is particularly pronounced in pathological states such as MDS [24]. Additionally, regulatory mechanisms such as hepcidin-mediated iron restriction and oxidative stress further impair hemoglobinization, contributing to ineffective erythropoiesis observed in these patients [26, 27].

### Dysregulated late erythropoiesis in LR-MDS

In LR-MDS, multiple disruptions of this finely tuned process become evident. Despite the elevated endogenous EPO levels, erythroid progenitors in LR-MDS frequently exhibit resistance to EPO stimulation. This resistance is attributed to the downregulation of EPOR and increased expression of suppressors of cytokine signaling proteins, such as SOCS1 and SOCS3, which blunt STAT5 phosphorylation and diminish the proliferative response to EPO [28]. Moreover, genetic alterations commonly seen in MDS, such as mutations in *SF3B1*, disrupt normal splicing and lead to increased degradation of SMAD7, thereby amplifying TGF-β signaling and further impeding erythroid maturation [29–31]. The bone marrow microenvironment in MDS is also characterized by heightened production of inflammatory cytokines, including interferon-gamma and tumor necrosis factor-alpha, which further suppress erythropoiesis and perpetuate anemia [32].

### Impaired telomere maintenance in LR-MDS

Telomere maintenance is essential for the function of hematopoietic stem cells (HSCs), and its dysregulation is a key feature of LR-MDS. In healthy hematopoiesis, telomerase activity preserves telomere length, supporting sustained cell division and genomic stability. However, in LR-MDS, clonal HSCs experience accelerated telomere shortening, often outpacing the compensatory capacity of telomerase [33, 34]. This imbalance leads to increased genomic instability, which promotes clonal selection and the accumulation of mutations that drive ineffective hematopoiesis and disease progression.

The pathophysiology of LR-MDS is further complicated by chronic inflammatory signals in the bone marrow microenvironment that intensify oxidative stress and contribute to telomere shortening. As telomeres shorten, the affected HSCs become increasingly prone to DNA damage, resulting in impaired erythropoiesis and persistent cytopenia. Patients with shorter telomeres have a higher risk of leukemic transformation and poorer survival [35–37], underscoring the clinical significance of telomere dysfunction in LR-MDS.

## Current therapies for anemia correction in LR-MDs: Erythropoiesis-Stimulating Agents (ESAs) and lenalidomide

### ESAs

ESAs, including epoetin alfa and darbepoetin alfa, remain the initial therapeutic choice for anemia in LR-MDS. These agents enhance erythropoiesis by activating the JAK2/STAT5 signaling pathway via EPOR binding. While ESAs achieve hematologic improvement in approximately 40% of patients, responses are often transient (median duration: 1.5–2 years) [38–41], with resistance developing owing to EPOR downregulation, SOCS1/3 overexpression, and clonal evolution [42]. Real-world studies corroborate these limitations, showing that fewer than 30% of transfusion-dependent patients achieve sustained transfusion independence (TI), whereas over 50% remain dependent on transfusions despite ESA therapy [43].

### Lenalidomide in del(5q) LR-MDS

Lenalidomide represents a significant advancement for patients with LR-MDS with an isolated chromosome 5q deletion (del[5q]). Its mechanism involves synthetic lethality, selectively targeting malignant clones through cereblon (CRBN)-mediated proteasomal degradation of casein kinase 1α (CK1α). This degradation stabilizes p53, inducing apoptosis in del(5q) cells with CSNK1A1 haploinsufficiency while sparing normal hematopoiesis [44]. Clinical trials have underscored the efficacy of lenalidomide; the MDS-003 trial reported a 67% TI rate with a median response duration of 2.2 years [45]. In the phase 3 MDS-004 study, 56.1% of patients in the lenalidomide 10 mg group achieved RBC-TI for ≥ 26 weeks compared with 5.9% in the placebo group. Furthermore, among cytogenetic responders to lenalidomide, an 87% 10-year overall survival estimate was observed, significantly outperforming the non-cytogenetic responders (4% 10-year survival estimate) and historical cohorts [46]. Long-term follow-up data strongly suggest that lenalidomide improves overall survival by reducing the transfusion burden and serum ferritin [47]. Myelosuppression (grade 3–4 neutropenia and thrombocytopenia in 55% and 44% of patients, respectively) can be managed with dose adjustments and monitoring [48]. Despite its success, the utility of lenalidomide is largely restricted to patients with del(5q) biomarkers.

## Luspatercept: a first-in-class erythroid maturation agent

Luspatercept is a recombinant fusion protein designed to modulate TGF-β superfamily signaling. Its mechanism involves binding to ligands such as GDF11 and activins via a modified extracellular domain of activin receptor type IIB (ActRIIB), effectively acting as a “ligand trap.” By inhibiting aberrant Smad2/3 signaling, luspatercept promotes late-stage erythroid maturation, addressing the ineffective erythropoiesis characteristics of MDS [49–51]. This is in contrast to ESAs, which act on early erythroid progenitors and require intact EPO signaling.

### Clinical evidence and approval in LR-MDS with ring sideroblasts or mutated *SF3B1*

The PACE-MDS study evaluated luspatercept in all patients with LR-MDS and demonstrated overall robust erythroid responses. Patients with ring sideroblasts (RS-positive) and *SF3B1* mutations exhibited significantly higher rates of HI-E (67.7% and 74.5%, respectively) and RBC-TI [52, 53]. These promising phase 2 findings were subsequently confirmed in the phase 3 MEDALIST trial, which specifically enrolled patients with transfusion-dependent LR-MDS and RS refractory to ESAs. MEDALIST demonstrated that luspatercept significantly increased the 8-week RBC-TI (38% vs. 13% for placebo; *P* < 0.001). Furthermore, a higher percentage of luspatercept-treated patients achieved 12-week RBC-TI (28% vs. 8% for weeks 1–24; *P* < 0.001) [19].

These results highlight the heightened sensitivity of RS-positive and *SF3B1*-mutated MDS to luspatercept, even when standard ESAs fail owing to elevated EPO levels. RS are abnormal erythroid precursors with iron-laden mitochondria [54], often caused by *SF3B1* mutations that disrupt RNA splicing and lead to the RS phenotype [55, 56]. The enhanced drug efficacy in these subgroups was attributed to a strong alignment between disease biology and drug mechanisms, explaining the superior and more durable responses observed. This led to the regulatory approval of luspatercept for patients with LR-MDS with RS or *SF3B1* mutations after ESA failure.

### Broader approval to whole LR-MDS

As positive results were observed in prior research on LR-MDS patients who did not have RS or *SF3B1* mutations, the COMMANDS phase III trial [20] expanded luspatercept use to all LR-MDS patients regardless of RS or SF3B1 status. In this first-line study, luspatercept outperformed ESAs, achieving a composite primary endpoint (RBC-TI ≥ 12 weeks plus Hb increase ≥ 1.5 g/dL) in 58.5% of patients versus 31.2% with ESAs. The median duration of response (DoR) was significantly longer with luspatercept at 2.5 years, compared with 1.5 years with ESAs, and Hb increases ≥ 1.5 g/dL occurred in 47.6% versus 29.2% of patients, respectively [20]. For responders, this translates to approximately one additional year of transfusion-free survival over ESAs, making luspatercept a highly valuable first-line option. Safety was consistent with previous data, with no new toxicity signals [20]. This led to the 2023 FDA approval of luspatercept as a frontline therapy for anemia in all patients with LR-MDS, marking a paradigm shift in treatment accessibility.

### Real-world evidence of luspatercept

Real-world data have consistently confirmed the efficacy and safety of luspatercept. In a large Italian multicenter study of 201 patients with LR-MDS and RS [57], 30.8% achieved RBC-TI for at least 8 weeks within the first 24 weeks, with responses most pronounced among those with a lower baseline transfusion burden; the median duration of transfusion independence was 23.9 weeks, and the safety profile was manageable even in a frail, older population. Belgian real-world data [58] in a cohort with a median age of 79 years found a similar 35.4% rate of RBC-TI ≥ 8 weeks, with an overall response rate of 65.8%. However, treatment discontinuation due to adverse events was somewhat higher than in clinical trials, reflecting the increased frailty of this population. Complementing these findings, a large U.S. claims-based analysis of 871 patients with MDS treated with luspatercept reported that 87.4% achieved or maintained 8-week transfusion independence or non-dependence within 6 months, and two-thirds required no additional MDS-directed therapy after luspatercept was initiated. RS-positive and RS-negative subgroups derived similar benefits [59].

## Imetelstat: a first-in-class telomerase inhibitor

Imetelstat is a 13-mer oligonucleotide that binds to the RNA template (TERC) of telomerase, thereby inhibiting telomere elongation in malignant hematopoietic stem and progenitor cells. By accelerating telomere shortening, imetelstat selectively induces apoptosis in clonal cells with high telomerase activity, while sparing normal hematopoiesis. This mechanism targets the replicative immortality of MDS clones, offering a novel approach to address anemia and underlying clonal dynamics [60].

### Clinical evidence

The IMerge phase III trial (NCT02598661) evaluated imetelstat in patients with transfusion-dependent LR-MDS refractory to or ineligible for ESAs. Imetelstat achieved RBC-TI for ≥ 8 weeks in 40% of patients compared with 15% with placebo, with a median DoR of 51.6 weeks versus 13.3 weeks in the placebo group [21]. Efficacy was consistent across subgroups, including patients, regardless of ring RS status, mutational profile (e.g., *SF3B1, TET2*), or baseline telomere length, suggesting broad applicability [21]. However, hematologic toxicity was notable, with grade 3–4 neutropenia and thrombocytopenia occurring in > 40% and > 20% of the patients, respectively. This cytopenia was transient and manageable through dose modifications, with no reported long-term sequelae [21].

### Potential for disease modification

Imetelstat has demonstrated intriguing signals of disease modification, including reduction in the variant allele frequency (VAF) of common mutations, such as *SF3B1* and *TET2*, in responders [21–23]. A post hoc analysis of IMerge showed a 60% lower risk of AML progression in responders (hazard ratio 0.4, *p* = 0.08), although statistical significance was not achieved [21]. However, definitive evidence for clonal hematopoietic regression or a sustained reduction in marrow blasts remains elusive. Although preclinical models support telomerase inhibition as a strategy to suppress clonal dominance, clinical translation requires further validation through long-term follow-up and correlative studies.

## Ongoing challenges in the era of multiple anemia-correction agents in LR-MDS

The availability of four anemia-correction agents (ESAs, lenalidomide, luspatercept, and imetelstat) has transformed the management of LR-MDS. However, critical challenges persist in optimizing their use.

First, although luspatercept and imetelstat have demonstrated efficacy in patients with ESA-refractory LR-MDS (Table 1), information regarding which agent may be more beneficial for specific patient subgroups is limited. Further research is needed to guide optimal patient selection. In the pivotal MEDALIST trial, luspatercept achieved RBC-TI in 38% of ESA-refractory or ineligible patients, with a median DoR of 30.6 weeks [19]. In the IMerge trial, imetelstat achieved RBC-TI in 40% of a similar population, with a median DoR of 51.6 weeks [21]. Luspatercept is administered subcutaneously every 3 weeks and is generally well tolerated, whereas imetelstat is administered intravenously every 4 weeks and is associated with transient but significant cytopenia. Both agents show efficacy across subgroups regardless of RS or *SF3B1* mutation status. However, luspatercept is now approved for all patients with LR-MDS, while the use of imetelstat is focused on those who are ESA-refractory or ineligible. Biomarkers such as *SF3B1* mutations or telomere length have not consistently predicted responses, highlighting the need for further prospective studies that directly compare these agents or clarify the optimal selection criteria.

**Table 1:** Results of pivotal phase 3 trials for luspatercept (MEDALIST) and imetelstat (IMerge) in erythropoiesis-stimulating agent-refractory or ineligible patients with lower-risk myelodysplastic syndrome

Parameters	Luspatercept (MEDALIST)	Imetelstat (IMerge)
Inclusion criteria	2–6 u of RBC TF in 8 weeks	≥ 4 u of RBC TF in 16 weeks (no upper TF limit)
Patient’s convenience	S.C. injection every 3 weeks	2 h I.V. injection every 4 weeks
RBC-TI ≥ 8 weeks	38% (vs. placebo 13%)	40% (vs. placebo 15%)
Median duration of response	30.6 weeks	51.6 weeks
Hb increase	> 1.5 g/dL	3.6 g/dL
Safety	Probably the safest drug	Thrombocytopenia and neutropenia (transient and manageable)
Potential for disease modification	Not likely (erythroid maturation)	Maybe (originally developed for anticancer drug)

*RBC* red blood cell, *TF* transfusion, *RBC-TI* red blood cell-transfusion independence, *S.C.* subcutaneous, *I.V.* intravenous, *Hb* hemoglobin

**Fig. 1:**
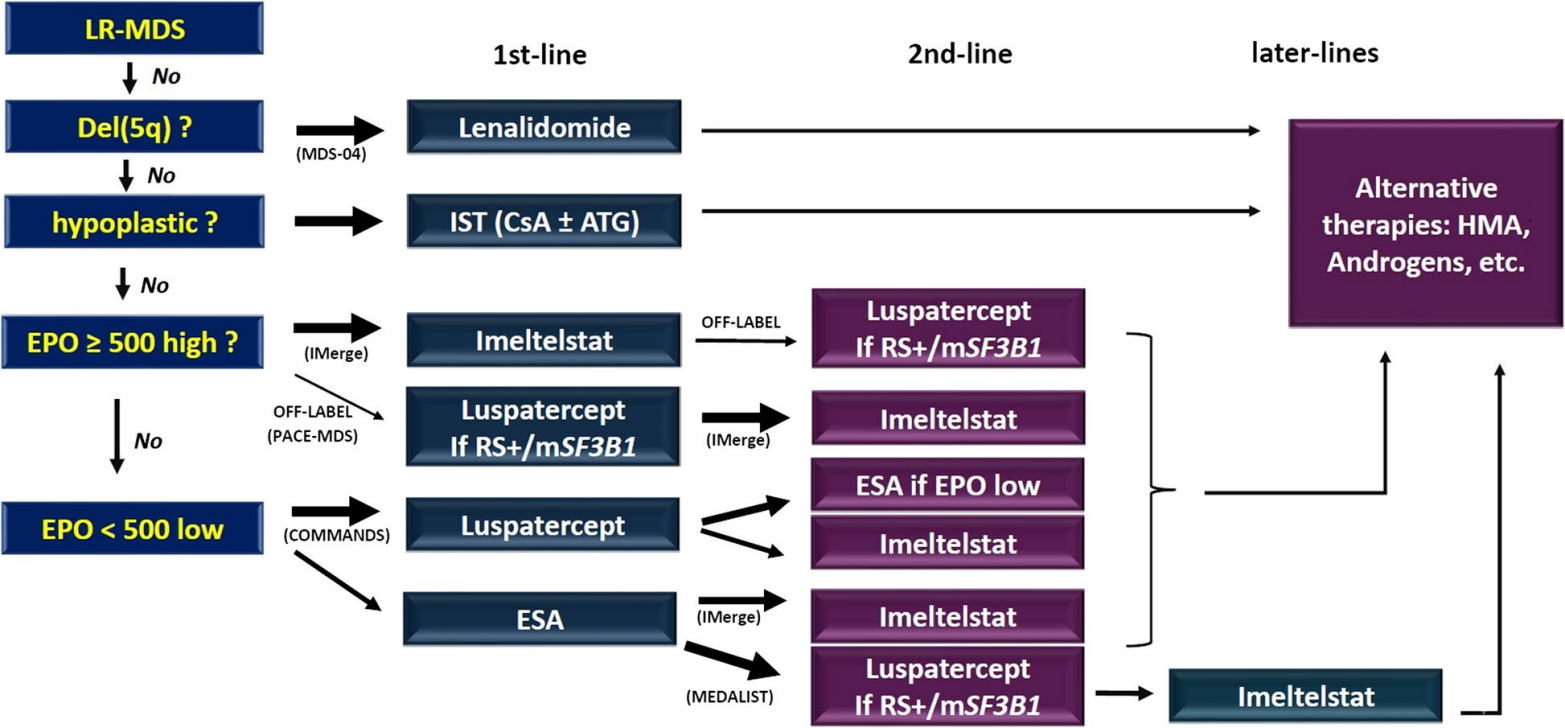
Possible sequential approach to anemia-correcting therapy in lower-risk myelodysplastic syndromes

Second, the optimal sequence or combination of these agents remains unclear (Fig. 1). ESAs are typically used as the first-line treatment in patients with serum EPO < 500 U/L, and lenalidomide is reserved for patients with del(5q). However, for ESA-refractory patients, the lack of robust biomarkers beyond RS/*SF3B1* status or baseline EPO levels complicates decisions regarding subsequent therapies. Current predictive tools do not adequately address factors such as clonal heterogeneity, inflammatory microenvironment signatures, or erythroid progenitor exhaustion, which may influence the treatment response. Prospective trials evaluating biomarker-driven algorithms and combination strategies are critical to resolving these uncertainties.

Finally, even among patients who achieve anemia correction, many with LR-MDS ultimately progress to high-risk MDS or acute myeloid leukemia. Imetelstat has shown reductions in the VAF of mutations, such as *SF3B1* and *TET2*, in responders, suggesting possible disease-modifying activity; however, definitive evidence for clonal regression or improved long-term outcomes remains lacking. Research into combining anemia-correction agents with disease-modifying therapies or developing new agents with proven impacts on disease progression remains a high priority.

## Conclusion

The introduction of luspatercept and imetelstat has expanded the treatment options for anemia in patients with lower-risk MDS, with luspatercept now established as a first-line standard, and imetelstat offering a valuable alternative for ESA-refractory patients. Both agents show efficacy across diverse clinical subgroups; however, the absence of predictive biomarkers and uncertainty regarding optimal sequencing remain key challenges. Although imetelstat demonstrates the potential for disease modification, further evidence is needed to confirm its long-term effects. In Korea, the lack of local introduction of imetelstat and reimbursement for luspatercept currently limits their clinical use, highlighting a critical area for improvement. Ongoing research should focus on individualized treatment strategies and long-term disease control based on molecular and clinical insights.

## Data Availability

No datasets were generated or analysed during the current study.

## References

[1] Choi HY, Chang JE. Targeted therapy for cancers: from ongoing clinical trials to FDA-approved drugs. Int J Mol Sci. 2023. 10.3390/ijms241713618. 37686423 10.3390/ijms241713618 PMC10487969

[2] Prasad V, Haslam A, Olivier T. Updated estimates of eligibility and response: immune checkpoint inhibitors. J Clin Oncol. 2024;42:e14613–e14613.

[3] Rajkumar SV. Multiple myeloma: 2022 update on diagnosis, risk stratification, and management. Am J Hematol. 2022;97:1086–107. 35560063 10.1002/ajh.26590 PMC9387011

[4] Tosato G, Feng JX, Ohnuki H, Sim M. Bone marrow niches in myelodysplastic syndromes. J Cancer Metastasis Treat. 2021;7:52. 34746416 10.20517/2394-4722.2021.120 PMC8570581

[5] Qin T, Castoro R, El Ahdab S, et al. Mechanisms of resistance to decitabine in the myelodysplastic syndrome. PLoS ONE. 2011;6:e23372. 21858090 10.1371/journal.pone.0023372 PMC3157379

[6] Kang SH, Choi JS. Microrna-765 is upregulated in myelodysplastic syndromes and induces apoptosis via PLP2 inhibition in leukemia cells. Blood Res. 2023;58:133–7. 37495419 10.5045/br.2023.2023097 PMC10548289

[7] Fenaux P, Mufti GJ, Hellstrom-Lindberg E, et al. Azacitidine prolongs overall survival compared with conventional care regimens in elderly patients with low bone marrow blast count acute myeloid leukemia. J Clin Oncol. 2010;28:562–9. 20026804 10.1200/JCO.2009.23.8329

[8] Yoo KH, Cho J, Han B, et al. Outcomes of decitabine treatment for newly diagnosed acute myeloid leukemia in older adults. PLoS ONE. 2020;15:e0235503. 32760083 10.1371/journal.pone.0235503 PMC7410295

[9] Venugopal S, Dinardo CD, Takahashi K, et al. Phase II study of the IDH2-inhibitor enasidenib in patients with high-risk IDH2-mutated myelodysplastic syndromes (MDS). J Clin Oncol. 2021;39:7010–7010.

[10] Garcia-Manero G, Griffiths EA, Steensma DP, et al. Oral cedazuridine/decitabine for MDS and CMML: a phase 2 pharmacokinetic/pharmacodynamic randomized crossover study. Blood. 2020;136:674–83. 32285126 10.1182/blood.2019004143 PMC7414597

[11] Kim H, Lee JY, Yu S, et al. Acute myeloid leukemia and myelodysplastic neoplasms: clinical implications of myelodysplasia-related genes mutations and TP53 aberrations. Blood Res. 2024;59:41. 39692933 10.1007/s44313-024-00044-4 PMC11655781

[12] Cho YU. The role of next-generation sequencing in hematologic malignancies. Blood Res. 2024;59:11. 38485897 10.1007/s44313-024-00010-0 PMC10917716

[13] Song JS. Clinically relevant core genes for hematologic malignancies in clinical NGS panel testing. Blood Res. 2023;58:224–8. 37926559 10.5045/br.2023.2023196 PMC10758630

[14] Liu J, Min S, Kim D, et al. Pharmacological GLUT3 salvage augments the efficacy of vitamin C-induced TET2 restoration in acute myeloid leukemia. Leukemia. 2023;37:1638–48. 37393342 10.1038/s41375-023-01954-5

[15] Papaemmanuil E, Gerstung M, Malcovati L, et al. Clinical and biological implications of driver mutations in myelodysplastic syndromes. Blood. 2013;122:3616–27 quiz 3699. 24030381 10.1182/blood-2013-08-518886 PMC3837510

[16] Malcovati L, Stevenson K, Papaemmanuil E, et al. SF3B1-mutant MDS as a distinct disease subtype: a proposal from the International Working Group for the Prognosis of MDS. Blood. 2020;136:157–70. 32347921 10.1182/blood.2020004850 PMC7362582

[17] Zeidan AM, Boss I, Beach CL, et al. A randomized phase 2 trial of azacitidine with or without durvalumab as first-line therapy for higher-risk myelodysplastic syndromes. Blood Adv. 2022;6:2207–18. 34972214 10.1182/bloodadvances.2021005487 PMC9006291

[18] Sallman DA, Al Malki MM, Asch AS, et al. Magrolimab in combination with azacitidine in patients with higher-risk myelodysplastic syndromes: final results of a phase Ib study. J Clin Oncol. 2023;41:2815–26. 36888930 10.1200/JCO.22.01794 PMC10414740

[19] Fenaux P, Platzbecker U, Mufti GJ, et al. Luspatercept in patients with lower-risk myelodysplastic syndromes. N Engl J Med. 2020;382:140–51. 31914241 10.1056/NEJMoa1908892

[20] Della Porta MG, Garcia-Manero G, Santini V, et al. Luspatercept versus epoetin alfa in erythropoiesis-stimulating agent-naive, transfusion-dependent, lower-risk myelodysplastic syndromes (COMMANDS): primary analysis of a phase 3, open-label, randomised, controlled trial. Lancet Haematol. 2024;11:e646–58. 39038479 10.1016/S2352-3026(24)00203-5

[21] Platzbecker U, Santini V, Fenaux P, et al. Imetelstat in patients with lower-risk myelodysplastic syndromes who have relapsed or are refractory to erythropoiesis-stimulating agents (IMerge): a multinational, randomised, double-blind, placebo-controlled, phase 3 trial. Lancet. 2024;403:249–60. 38048786 10.1016/S0140-6736(23)01724-5

[22] Majidi F, Gattermann N. Imetelstat: finally a disease-modifying treatment for lower-risk myelodysplastic syndromes? Med. 2024;5:184–6. 38460498 10.1016/j.medj.2024.01.004

[23] Santini V, Platzbecker U, Fenaux P, et al. MDS-605 disease modifying activity of imetelstat in patients with heavily transfused non-del(5q) lower-risk myelodysplastic syndromes relapsed/refractory/ineligible for erythropoiesis-stimulating agents in IMerge phase 3. Clin Lymphoma Myeloma Leuk. 2023;23:S373–4.

[24] Zermati Y, Fichelson S, Valensi F, et al. Transforming growth factor inhibits erythropoiesis by blocking proliferation and accelerating differentiation of erythroid progenitors. Exp Hematol. 2000;28(8):885–94. 10989189 10.1016/s0301-472x(00)00488-4

[25] Caulier AL, Sankaran VG. Molecular and cellular mechanisms that regulate human erythropoiesis. Blood. 2022;139:2450–9. 34936695 10.1182/blood.2021011044 PMC9029096

[26] Camaschella C, Nai A. Ineffective erythropoiesis and regulation of iron status in iron loading anaemias. Br J Haematol. 2016;172:512–23. 26491866 10.1111/bjh.13820

[27] Koh JS, Song IC. Functional iron deficiency anemia in patients with cancer. Blood Res. 2024;59:26. 39110268 10.1007/s44313-024-00030-w PMC11306885

[28] Hellstrom-Lindberg E, Negrin R, Stein R, et al. Erythroid response to treatment with G-CSF plus erythropoietin for the anaemia of patients with myelodysplastic syndromes: proposal for a predictive model. Br J Haematol. 1997;99:344–51. 9375752 10.1046/j.1365-2141.1997.4013211.x

[29] De La Garza A, Cameron RC, Gupta V, et al. The splicing factor Sf3b1 regulates erythroid maturation and proliferation via TGFbeta signaling in zebrafish. Blood Adv. 2019;3:2093–104. 31300417 10.1182/bloodadvances.2018027714 PMC6650725

[30] Zhou L, McMahon C, Bhagat T, et al. Reduced SMAD7 leads to overactivation of TGF-beta signaling in MDS that can be reversed by a specific inhibitor of TGF-beta receptor I kinase. Cancer Res. 2011;71:955–63. 21189329 10.1158/0008-5472.CAN-10-2933 PMC3032816

[31] Jiang M, Chen M, Liu Q, et al. SF3B1 mutations in myelodysplastic syndromes: a potential therapeutic target for modulating the entire disease process. Front Oncol. 2023;13:1116438. 37007111 10.3389/fonc.2023.1116438 PMC10063959

[32] Sallman DA, List A. The central role of inflammatory signaling in the pathogenesis of myelodysplastic syndromes. Blood. 2019;133:1039–48. 30670444 10.1182/blood-2018-10-844654 PMC7022316

[33] Park HS, Choi J, See CJ, et al. Dysregulation of telomere lengths and telomerase activity in myelodysplastic syndrome. Ann Lab Med. 2017;37:195–203. 28224765 10.3343/alm.2017.37.3.195 PMC5339091

[34] Gurkan E, Tanriverdi K, Baslamisli F. Telomerase activity in myelodysplastic syndromes. Leuk Res. 2005;29:1131–9. 16111531 10.1016/j.leukres.2005.03.006

[35] Hwang SM, Kim SY, Kim JA, et al. Short telomere length and its correlation with gene mutations in myelodysplastic syndrome. J Hematol Oncol. 2016;9:62. 27465399 10.1186/s13045-016-0287-9 PMC4964031

[36] Göhring G, Lange K, Hofmann W, et al. Short telomeres before lenalidomide treatment predict leukemic progression in patients with myelodysplastic syndrome and deletion 5q. Blood. 2010;116:30.

[37] Myllymaki M, Redd R, Reilly CR, et al. Short telomere length predicts nonrelapse mortality after stem cell transplantation for myelodysplastic syndrome. Blood. 2020;136:3070–81. 33367544 10.1182/blood.2020005397 PMC7770569

[38] Hellstrom-Lindberg E, Ahlgren T, Beguin Y, et al. Treatment of anemia in myelodysplastic syndromes with granulocyte colony-stimulating factor plus erythropoietin: results from a randomized phase II study and long-term follow-up of 71 patients. Blood. 1998;92:68–75. 9639501

[39] Jadersten M, Malcovati L, Dybedal I, et al. Erythropoietin and granulocyte-colony stimulating factor treatment associated with improved survival in myelodysplastic syndrome. J Clin Oncol. 2008;26:3607–13. 18559873 10.1200/JCO.2007.15.4906

[40] Park S, Grabar S, Kelaidi C, et al. Predictive factors of response and survival in myelodysplastic syndrome treated with erythropoietin and G-CSF: the GFM experience. Blood. 2008;111:574–82. 17940203 10.1182/blood-2007-06-096370

[41] Platzbecker U, Symeonidis A, Oliva EN, et al. A phase 3 randomized placebo-controlled trial of darbepoetin alfa in patients with anemia and lower-risk myelodysplastic syndromes. Leukemia. 2017;31:1944–50. 28626220 10.1038/leu.2017.192 PMC5596208

[42] Kubasch AS, Platzbecker U. Setting fire to ESA and EMA resistance: new targeted treatment options in lower risk myelodysplastic syndromes. Int J Mol Sci. 2019. 10.3390/ijms20163853. 31394818 10.3390/ijms20163853 PMC6720617

[43] Diez-Campelo M, Yucel A, Goyal RK, et al. Treatment characteristics and outcomes in lower-risk, non-del(5q) myelodysplastic syndromes: findings from a medical record review in the USA, Canada and Europe. Future Oncol. 2024;20:1993–2004. 39140298 10.1080/14796694.2024.2379228 PMC11497946

[44] Fink EC, Ebert BL. The novel mechanism of lenalidomide activity. Blood. 2015;126:2366–9. 26438514 10.1182/blood-2015-07-567958 PMC4653765

[45] List A, Dewald G, Bennett J, et al. Lenalidomide in the myelodysplastic syndrome with chromosome 5q deletion. N Engl J Med. 2006;355:1456–65. 17021321 10.1056/NEJMoa061292

[46] Fenaux P, Giagounidis A, Selleslag D, et al. A randomized phase 3 study of lenalidomide versus placebo in RBC transfusion-dependent patients with low-/intermediate-1-risk myelodysplastic syndromes with del5q. Blood. 2011;118:3765–76. 21753188 10.1182/blood-2011-01-330126

[47] Santini V, Giagounidis A, Pelligra CG, et al. Impact of lenalidomide treatment on overall survival in patients with lower-risk, transfusion-dependent myelodysplastic syndromes. Clin Lymphoma Myeloma Leuk. 2022;22:e874–83. 35710702 10.1016/j.clml.2022.05.001

[48] Hong J, Lee YJ, Bae SH, et al. Lenalidomide for anemia correction in lower-risk del(5q) myelodysplastic syndrome patients of Asian ethnicity. Blood Res. 2021;56:102–8. 34187943 10.5045/br.2021.2021086 PMC8246035

[49] Attie KM, Allison MJ, McClure T, et al. A phase 1 study of ACE-536, a regulator of erythroid differentiation, in healthy volunteers. Am J Hematol. 2014;89:766–70. 24715706 10.1002/ajh.23732 PMC4173124

[50] Suragani RN, Cadena SM, Cawley SM, et al. Transforming growth factor-beta superfamily ligand trap ACE-536 corrects anemia by promoting late-stage erythropoiesis. Nat Med. 2014;20:408–14. 24658078 10.1038/nm.3512

[51] Suragani RN, Cawley SM, Li R, et al. Modified activin receptor IIB ligand trap mitigates ineffective erythropoiesis and disease complications in murine beta-thalassemia. Blood. 2014;123:3864–72. 24795345 10.1182/blood-2013-06-511238 PMC4064330

[52] Platzbecker U, Gotze KS, Kiewe P, et al. Long-term efficacy and safety of luspatercept for anemia treatment in patients with lower-risk myelodysplastic syndromes: the phase II PACE-MDS study. J Clin Oncol. 2022;40:3800–7. 35998303 10.1200/JCO.21.02476 PMC9671752

[53] Platzbecker U, Germing U, Gotze KS, et al. Luspatercept for the treatment of anaemia in patients with lower-risk myelodysplastic syndromes (PACE-MDS): a multicentre, open-label phase 2 dose-finding study with long-term extension study. Lancet Oncol. 2017;18:1338–47. 28870615 10.1016/S1470-2045(17)30615-0

[54] Fontenay M, Cathelin S, Amiot M, Gyan E, Solary E. Mitochondria in hematopoiesis and hematological diseases. Oncogene. 2006;25:4757–67. 16892088 10.1038/sj.onc.1209606

[55] Ochi T, Fujiwara T, Ono K, et al. Exploring the mechanistic link between SF3B1 mutation and ring sideroblast formation in myelodysplastic syndrome. Sci Rep. 2022;12:14562. 36028755 10.1038/s41598-022-18921-2 PMC9418223

[56] Malcovati L, Karimi M, Papaemmanuil E, et al. SF3B1 mutation identifies a distinct subset of myelodysplastic syndrome with ring sideroblasts. Blood. 2015;126:233–41. 25957392 10.1182/blood-2015-03-633537 PMC4528082

[57] Lanino L, Restuccia F, Perego A, et al. Real-world efficacy and safety of luspatercept and predictive factors of response in patients with lower risk myelodysplastic syndromes with ring sideroblasts. Am J Hematol. 2023;98:E204–8. 37222267 10.1002/ajh.26960

[58] Heyrman B, Meers S, Sid S, et al. Real-life data of luspatercept in lower-risk myelodysplastic syndromes advocate new research objectives. EJHaem. 2024;5:1096–9. 39415916 10.1002/jha2.1027 PMC11474394

[59] Andritsos LA, McBride A, Tang D, et al. Real-world impact of luspatercept on red blood cell transfusions among patients with myelodysplastic syndromes: a United States healthcare claims database study. Leuk Res. 2025;148:107624. 39602855 10.1016/j.leukres.2024.107624

[60] Keam SJ. Imetelstat: first approval. Drugs. 2024;84:1149–55. 39162963 10.1007/s40265-024-02080-x

